# Recovery for all in the community; position paper on principles and key elements of community-based mental health care

**DOI:** 10.1186/s12888-019-2162-z

**Published:** 2019-06-10

**Authors:** René Keet, Marjonneke de Vetten-Mc Mahon, Laura Shields-Zeeman, Torleif Ruud, Jaap van Weeghel, Michiel Bahler, Cornelis L. Mulder, Catherine van Zelst, Billy Murphy, Koen Westen, Chris Nas, Ionela Petrea, Guido Pieters

**Affiliations:** 1 GGZ NHN, Stationsplein 138, Heerhugowaard, The Netherlands; 2 Department Trimbos International, Trimbos Institute, Da Costakade 45, Utrecht, The Netherlands; 3 Clinical of Health Services Research and Psychiatry, Institute of Clinical Medicine, Medical Faculty, University of Oslo, Blindern, 0318 Oslo, Norway; 4 Mental Health Services, Akershus University Hospital, Lørenskog, Norway; 5 Kenniscentrum Phrenos, Da Costakade 45, Utrecht, The Netherlands; 6 Tranzo Scientific Center for Care and Welfare, Tilburg School of Social and Behavioural Sciences, Tilburg University, Tilburg, The Netherlands; 7 Department of Psychiatry, Erasmus MC, University Medical Center Rotterdam, Doctor Molewaterplein 40, Rotterdam, The Netherlands; 8 Department of Psychiatry and Neuropsychology, Maastricht University, P.O. Box 616, 6200 MD Maastricht, The Netherlands; 9 Inspire Mental Health, 10-20 Lombard Street, Belfast, Northern Ireland; 10 Nursing Department, Avans Hogeschool, Onderwijsboulevard 215, ‘s-Hertogenbosch, The Netherlands; 11 Zilveren Kruis Achmea, Dellaertweg 1, 2316 WZ Leiden, The Netherlands; 12 Department of Psychiatry, KU Leuven, UZ Herestraat 49, Leuven, Belgium

**Keywords:** Community-based mental health care, Position paper, Mental health system, Principles

## Abstract

**Background:**

Service providers throughout Europe have identified the need to define how high-quality community-based mental health care looks to organize their own services and to inform governments, commissioners and funders. In 2016, representatives of mental health care service providers, networks, umbrella organizations and knowledge institutes in Europe came together to establish the European Community Mental Health Services Provider (EUCOMS) Network. This network developed a shared vision on the principles and key elements of community mental health care in different contexts. The result is a comprehensive consensus paper, of which this position paper is an outline.

With this paper the network wants to contribute to the discussion on how to improve structures in mental healthcare, and to narrow the gap between evidence, policy and practice in Europe.

**Main text:**

The development of the consensus paper started with an expert workshop in April 2016. An assigned writing group representing the workshop participants built upon the outcomes of this meeting and developed the consensus paper with the input from 100 European counterparts through two additional work groups, and two structured feedback rounds via email.

High quality community-based mental health care: 1) protects human rights; 2) has a public health focus; 3) supports service users in their recovery journey; 4) makes use of effective interventions based on evidence and client goals; 5) promotes a wide network of support in the community and; 6) makes use of peer expertise in service design and delivery. Each principle is illustrated with good practices from European service providers that are members of the EUCOMS Network.

**Conclusions:**

Discussion among EUCOMS network members resulted in a blueprint for a regional model of integrated mental health care based upon six principles.

## Background

In Europe a wide range of community-based mental health care services have been implemented and the number of inpatient beds has decreased [1]. The combination of a change in attitude among service users and providers, the development of supporting policies and the growing knowledge on how community mental health care should be organized has contributed to this shift. However, this trend is not uniform and linear across Europe [1–7].

De-institutionalization and the development of community-based care has been an important mental health policy goal for over forty years [4]. Long-stay psychiatric hospitals have been losing their central role in mental health systems. However, these hospitals remain predominant in many countries, still consuming the majority of resources allocated to mental health [6].

In the transfer to community-based care the largest barriers include low political priority, and insufficient and inadequate funding. This is followed by a lack of consensus among stakeholders and cooperation between health and social sectors, difficulties with integrating mental health into primary health care, the lack of clear or strong leadership, and resistance to change [1].

To overcome these barriers advocacy strategies should be developed involving consumers, key decision-makers and organizations to create agreement on the course of action and political commitment [1, 2, 8, 9]. Subsequently policies, legislation and plans should be developed and updated to facilitate resource allocation to community-based mental health care services and ongoing collaboration between mental health, social and employment services [1, 2].

Despite European service providers playing an essential role in consensus building, advocacy and implementation, they have not been organized and structurally represented in the European policy arena or considered as a key stakeholder in the development and implementation of mental health policy.

In an effort to fill this gap, EUCOMS was established in 2015 during a meeting of the European Assertive Outreach Foundation (https://www.eaof.org). After this meeting the shared ‘values and ambitions’ of the network were drafted. This document was discussed with 30 experts in Europe to see if they align with their view on the scope of the network. From the interviews and the subsequent first network meeting in 2016 it was concluded that deinstitutionalization only defines what mental health systems need to leave behind. Key adverse effects of deinstitutionalisation without a clear defined alternative are homelessness and re/trans-institutionalization [10–13]. Therefore, EUCOMS expressed the need to define a shared vision on what ‘good’ community-based mental health care entails as an alternative, to organize their own services and to inform governments, commissioners and funders.

### Aim

This paper describes a shared vision in the form of a position paper outlining six principles underpinning the organization of good community-based mental health care in a distinct geographically defined region or catchment area. The practical implications of these principles have been illustrated with good practices from European service providers that are members of the EUCOMS Network. With this shared vision EUCOMS aims to contribute to the discussion on how to narrow the gap between evidence, policy and practice in Europe supporting the regional implementation of quality community mental health care taking into account the diverse contexts. The main question addressed in the position paper is: “what are the principles and key elements of high-quality community-based mental health care according to members of the EUCOMS network?”

## Methods

**Fig. 1:**
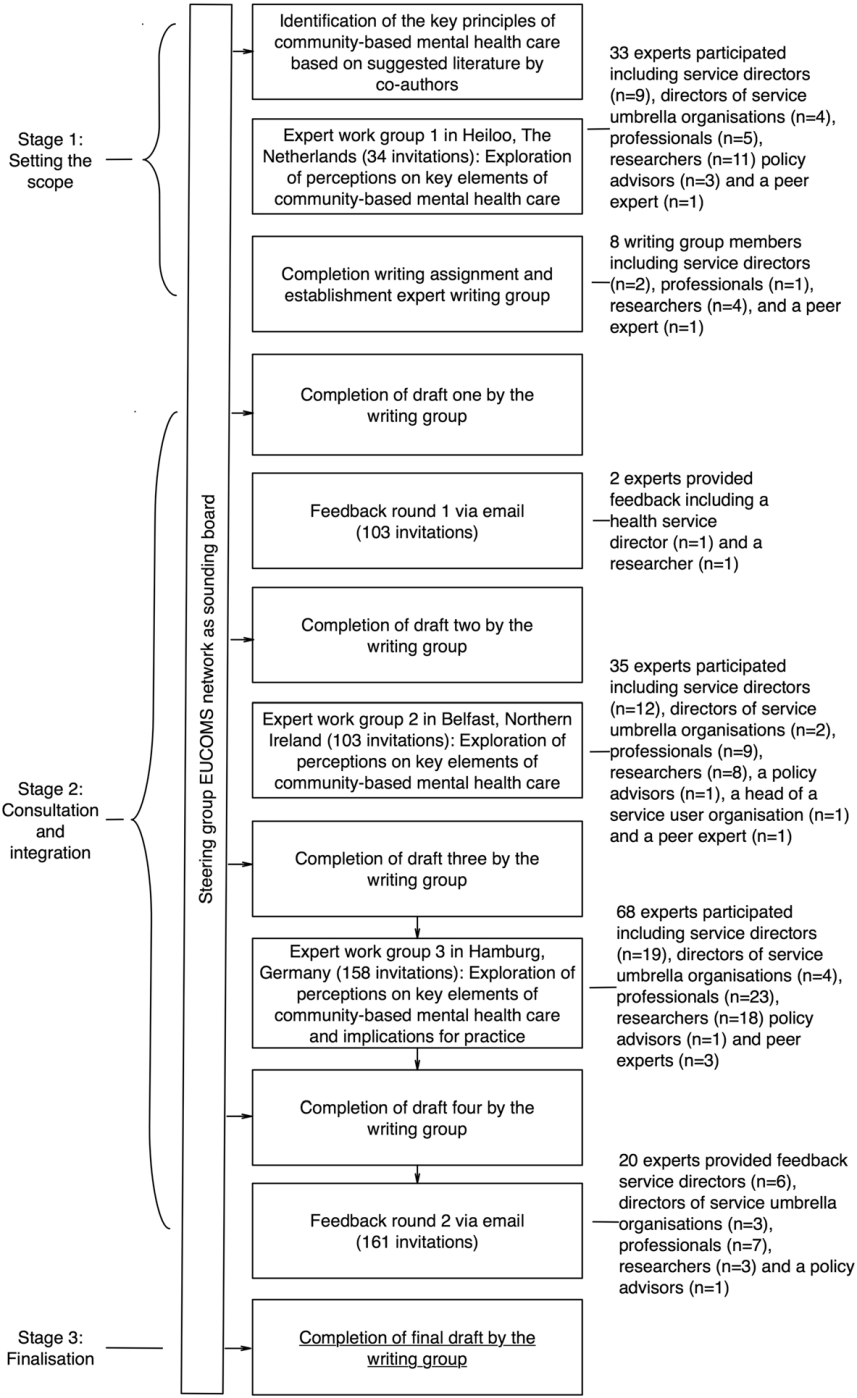
Development process of the position paper. The figure shows the stages the position paper was developed in, and the experts who have been involved in each stage

The position paper is based on professional-, scientific- and peer-expertise and has been developed between April 2016 and December 2017 in consultation with EUCOMS Network members, hereafter called experts, including mental health service directors, umbrella organisation directors, mental health care professionals, peer experts, researchers and policy advisors from nineteen different countries and a variety of European regions including Scandinavia, the British Isles, Western, Southern, Central, Eastern Europe, and the United States. The development process of the position paper is described in Fig. 1. This process description is in part ‘reconstructed logic’. Table 1 provides an overview of the number of work group participants and feedback respondents and their country of origin.

**Table 1:** Overview work group participants and feedback respondents and their country of origin

Country	Work Group Participants	Feedback Respondents
Austria	1	/
Belgium	6	/
Croatia	1	1
Czech Republic	6	1
France	2	1
Germany	21	5
Greece	2	1
Ireland	1	/
Italy	2	1
Luxembourg	1	2
Moldova	1	/
Montenegro	3	/
Netherlands	27	7
Norway	2	/
Romania	1	/
Spain	3	3
Switzerland	1	/
United Kingdom	9	1
United States	2	/
Total	92	21

### Stage 1: setting the scope

Co-authors suggested essential literature to define the main principles of quality community-based mental health care resulting in six principles of which the first four were based on the definition of Drake et al. (2011): “Community mental health care comprises the principles and practices needed to promote mental health for a local population by: 1) addressing population-based needs in ways that are accessible and acceptable; 2) building on the goals and strengths of people who experience mental illness; 3) promoting a wide network of supports, services, and resources of adequate capacity; and 4) emphasizing services that are both evidence-based and recovery-oriented.” Two additional principles were added based on the WHO mental health action plan (2013–2020) [14] and the results of the ‘Joint Action on Mental Health and Wellbeing’ [1]: 5) involving service users as co-creator in community-based mental health and; 6) providing assertive outreach.

Each of the six principles were discussed in the first expert work group of 2 h at Heiloo, The Netherlands in April 2016 based on the European Foundation for Quality Management (EFQM) framework [15]. Main questions addressed during the work group discussing each of the principles were “what is the desirable result we want to achieve from the perspective of the service user” and “what are the necessary elements of community-based mental health care to address these needs of the service user?”. The outcomes provided the scope for the consensus paper. In July 2016, a writing group was established to lead the writing process.

### Stage 2: consultation and integration

Expert consultations on the content of the consensus paper took place in the form of two work groups of 2 h, and two feedback rounds via email.

In January 2017 a draft of the position paper was discussed in six groups and a plenary session in a workshop. Each of the groups discussed one chapter covering one of the principles. In September 2017 the last expert workshop, with 68 participants focused on how each principle could be translated into practice proposing good practices.

In the first feedback round via email in December 2016, all EUCOMS contacts were asked to provide general feedback on the document. In the second feedback round, in September 2017, more in-depth comments on the position paper were requested through a feedback form where people could propose additions/changes per page.

### Stage 3: finalisation

The results of each consultation activity were integrated by the writing group reflecting the perspectives of the work group participants and the feedback respondents. After completion of the first, third, and final draft, feedback was requested from the steering group of EUCOMS to check whether the document was in line with their knowledge and expertise on key principles and elements of community-based mental health care. The comments received as a result of the last feedback round were structurally addressed by three of the writing group members. The final document was completed in October 2017 [16]. This position paper gives an outline of the Consensus Paper.

## Principles and key elements of community-based mental health care

This position paper conceptualised health as the dynamic ability to adapt and self-manage one’s own well-being to address the physical, emotional and social challenges of life [17]. This definition shifts the emphasis from ill-health to resilience and well-being stressing that the focus of community-based mental health care is on the promotion of mental health, integrating cure, care and prevention of mental illness. The position paper describes what high quality community mental health care looks based on six principles each outlined in a paragraph below:

1. Human rights
2. Public health
3. Recovery
4. Effectiveness of interventions
5. Community network of care
6. Peer expertise

### Human rights

After the second world war and the adoption of the Universal Declaration of Human Rights (UDHR) 1948 [18], the first steps were taken to close down psychiatric hospitals starting the process of transitioning mental health care to the community. Historically, the protection of human rights is one of the drivers for deinstitutionalization. Therefore, human rights represent the first principle and underpins all other elements of good community-based mental health care outlined in this position paper.

Goffman, in his landmark publication from 1961 ‘Asylums, Essays on the Social Situation of Mental Patients and other Inmates’ [19] questioned the need to keep people in institutions and the use of power and coercion in these institutions, rather serving and protecting the wider system than the inhabitants. This book reflected a change in thinking about the role of institutions providing mental health care and was a cornerstone for greater emphasis on human rights, participation in the community and the right to refuse treatment.

Although all population groups fall under the protection of human rights, certain groups experience more violations than others, including people with mental illness [20]. They suffer from stigma and discrimination resulting in exclusion from society and the inability to enact their right to liberty and security as well as to live independently included in the community, on an equal basis with others.

Landmark conventions responding to the need to assess and promote the human rights and fundamental freedoms of particular groups of people were the UN-Convention on the Elimination of All Forms of Discrimination against Women in 1979 [21], the Convention against Torture and Other Cruel, Inhuman or Degrading Treatment or Punishment (1984) [22] and UN-Convention on the Rights of the Child in 1989 [23].

In 2006, the human rights of people with disabilities were explicitly formulated in the Convention on the Rights of Persons with Disabilities (CRPD). In 2007 82 countries, including many European countries, signed this document reaffirming that persons with all types of disabilities must enjoy the same human rights and fundamental freedoms as anybody else [24]. In 2008, the CRPD came into force recognizing full and effective participation and inclusion in society as a general principle, obligation and right [25].

Although progress has been made in the implementation of the CRPD in Europe, there is still much to be done [4, 5, 25]. The life expectancy of people with severe mental illness has been estimated to be 15–25 years shorter than that of the general population, and there are indications that this mortality gap is widening [26–29]. This discrepancy can be explained by persisting stigma, discrimination and exclusion that affects their socio-economic position, help-seeking behaviour and the quality of care they receive [29].

The UN Report of the Special Rapporteur on the right of everyone to the enjoyment of the highest attainable standard of physical and mental health (2017) states that: ‘reductive biomedical approaches to treatment that do not adequately address contexts and relationships can no longer be considered compliant with the right to health’ [30].

To align with the standpoint of the Special Rapporteur and the CRPD the position paper recommends preventing exclusion from community life which negatively impacts the ability to integrate in society, achieve recovery goals, and lead a meaningful life. To realize this, governments need to ensure that the rights of all people are respected on the same legal basis. This requires a revision of national policies and legislative frameworks.

On a service level, it is recommended to base the mission and vision of the mental health service on the CRPD (2007) [24]. In addition, providing training and coaching to health and social care staff on recovery and rights can reduce human rights violations that occur in the context of mental health services.

One example of a tool to use to structurally improve mental health facilities is the Quality Rights Toolkit of the WHO, which offers a training framework for assessing and improving quality and human rights standards in mental health and social care services in line with the CRPD [31].

### Public health

Public health actions seek to achieve equity between groups and a state of population-level health [32], responding to the extended human right to “the highest attainable standard of health” [33]. This implies that society and services have a focus on the needs of the entire population. The mission of a good community mental health service is supporting the health of all citizens in their catchment area. This means that the focus is not only on treatment, but on mental health promotion and prevention as well, therefore taking the needs of the entire population into account, not only those with an existing mental health problem who seek help [34]. The society also benefits of good functioning mental health care. Mental health is a global public good and is relevant to sustainable development in all countries regardless of their socioeconomic status [35].

In most European countries there is insufficient focus on the public health function of mental health care services [36, 37]. Therefore, the position paper recommends taking a public health approach in the planning and implementation of community-based mental health care. This involves defining the catchment area for services and performing a needs assessment [38] and translating it into well-defined plans mapping services and supports for different segments of the population.

The size of the region depends on the regional demography, prevalence of mental ill health and the available resources for (mental) health care. It is primarily determined by two factors. First the region must be small enough for the team(s) to be integrated in the local community and have a strong relationship with primary care and social stakeholders. On the other hand, the region must be large enough to mobilize resources to build a multidisciplinary team in that region. Therefore, the size of the catchment area is a trade-off between the advantages of a small region (presence, collaboration with a small number of family doctors) and the necessity of sufficient resources to form a multidisciplinary team.

Secondly, the task of community-based mental health care services is prevention and treatment of mental health problems of citizens in the particular catchment area. This includes the employment of targeted interventions promoting social contact and anti-discrimination messages that can reduce stigma and enhance integration of people with mental ill health [29, 39]. Currently, well-known anti-stigma strategies include education (challenging myths with facts about mental health conditions) and contact (planned exchanges between people with lived experience and the general public). Corrigan et al. showed that programmes that are contact-based and provide education given by persons with lived experience, have better outcomes [40, 41].

Based on their studies Corrigan and his colleagues [42] constructed the TLC3 formula: Targeted, Local, Contact, Credible and Continuous. Examples of successful anti-stigma programs according to the TLC3 formula are the ‘Time to Change’ campaign in England, ‘One of Us’ campaign in Denmark, and the ‘SeeMe’ campaign in Scotland.

### Recovery

The most widely used definition describes recovery as “a deeply personal, unique process of changing one’s attitudes, values, feelings, goals, skills, and/or roles. It is a way of living a satisfying, hopeful, and contributing life even within the limitations caused by illness. Recovery involves the development of new meaning and purpose in one’s life as one grows beyond the catastrophic effects of mental illness” [43].

Recovery has several dimensions, including clinical recovery (relief of psychiatric symptoms); functional recovery (meaningful participation in society) and personal recovery (restoring personal identity). Recovery is a unique individual process or experience that may best be described as a journey [44–46]. In good community-based mental health care, professionals are companions on this journey for as short a time as possible but as long as necessary [47].

Over the past few years, a shift has taken place among many service providers in Europe aiming to realize individual needs and personal goals from the user’s perspective, with emphasis on autonomy and decision-making power of the client [2].

However, on some occasions, recovery has been used as window dressing. Though the concept of recovery is increasingly applied in mental health services, its actual application in practice is difficult to demonstrate [45]. It needs to be emphasized, therefore, that simply re-branding a service as ‘recovery-oriented’ or incorporating the word recovery in a logo or mission statement does not necessarily mean practicing recovery-oriented care.

To further support the recovery perspective, community-based mental health care services shift their focus from merely treatment to supporting people in their recovery journey. The consensus paper states principles that can help the professional to serve as a guide on the journey to recovery [16]. Important principles are: offer hope; decide with and not about the service user; focus on what is strong, and not on what is wrong.

### Effectiveness of interventions

Good community mental health care uses evidence-based interventions with effects that are documented with high-quality scientific evidence as shown in clinical guidelines (like the NICE guidelines) and systematic reviews and meta-analyses (like Cochrane reviews) [48, 49]. However, models of service organisation have scarcely been studied. New insights from implementation research can support the process of translating science to practice [50].

Evidence-based interventions and service delivery models cannot facilitate recovery in isolation. Scientific evidence is one of the important factors in making mental health care decisions. The context of the individual patient is essential in successfully realizing a treatment plan taking along the service users’ values, preferences, and choices. As community mental health is provided within one’s own natural environment, context-driven adaptations of evidence-based interventions are important to reflect local realities and resources. Therefore, evidence-based medicine and the recovery attitude go together like oil and vinegar: two approaches that can be combined very well and together make a tasty vinaigrette [51].

Historically, the development of the mental health field can be viewed in three waves or eras [52]. The first era was concerned with professional dominance and self-regulation. Era two, the current era, is the period for evidence-based medicine, accountability and market theory. The third and upcoming era in mental health is the moral era, with a reduction of mandatory measurements, giving up the professional prerogative, and a transition to civility and collaboration with patients and carers. The move towards the third era is driven by limited evidence of improved outcomes of biological and psychological approaches alone in mental healthcare [53, 54], and the growing knowledge on the powerful influence of social factors, like inequality in mental health [55, 56].

In Europe evidence-based approaches and recovery oriented care have gone hand-in-hand in the recent years [51]. However, the risk remains of divided camps between evidence-based and recovery-oriented care.

To support the move towards the third era EUCOMS recommends the use of interventions that integrate a focus on evidence-based psychological treatment (such as cognitive behavioural therapy (CBT) [57], motivational interviewing (MI) [58], and psychodynamic therapy [59–61]) with the use of medication as a tool and not as an aim. Furthermore, there is a focus on improving physical health and social inclusion. Best practices that focus on social inclusion include the use of resource groups [62], dialogue approaches [63], housing first [64] and individual placement and support (IPS) [65].

### Community network of care

Like a beehive, community mental health is a network that operates within a broader network of self-help, family, friends and other informal resources and generic community services [66]. Cross pollination is a symbol for collaboration between these different parties. The aim of this combination of perspectives in care is to bridge the gap between professionals and non-professionals, in order to increase the resilience of users as well as the resilience of the networks around users. Thus, mental health becomes an issue of multiple collaborating providers with a whole system approach [67].

Community-based mental health services typically consist of a multidisciplinary, multi-service therapeutic care network that can provide a broad spectrum of flexible interventions tailored to needs of users, which will ultimately allow people to recover in their home environment with support from their social network. Depending on the resources available, care can be provided through integrated care models with a community mental health care team as the central node, or by separate teams or functions of more generic teams if integrated care is not available. Whatever choice is made here, primary care is the main network partner in the medical domain. Therefore EUCOMS is operating together with the working group of the European Forum for Primary Care (EFPC) [68].

It is important that the different disciplines in a community mental health team take a shared responsibility for the interventions. This implies an interdisciplinary and a multi-expert way of working in which there are no exclusive domains. Expertise varies per discipline, and it is a task of the experts in the team to share their expertise, e.g. by organizing clinical lessons. Furthermore, the professional expertise of team members is combined with the lived experience of users. The same principle can be applied for intersectoral collaboration [69].

In Europe many models of integrated community mental health care have been implemented, where a range of health services work together to provide customized care. Assertive community treatment (ACT) is an evidence based integrated community-based mental health care model. ACT has shown to offer significant advantages over standard case management models in reducing homelessness and symptom severity in homeless persons with severe mental illness [70]. Good practices of integrated community-based mental health care with less conclusive evidence include; Flexible Assertive Community Treatment (F-ACT) [71, 72], and integrated dual disorder treatment (IDDT) [73, 74].

The collaboration with care beyond the mental health care system such as relatives and social care is underdeveloped and needs more attention [1, 75–77]. An opportunity for empowerment for persons living with mental ill health is becoming member of a Clubhouse [78]. During the course of their participation in a Clubhouse, members gain access to opportunities to re-join the worlds of friendships, family, employment and education, and to the services and support they may individually need beyond the mental health care system to continue their recovery [79].

The integration of the community mental health care services, sectors and collaboration with the social network of the service user can be hindered by a financing system that favours institutional care (e.g. by rewarding bed occupation) [36, 80]. Therefore, it is recommended to create a flexible financing system that allows incentives for different services that address the relevant life domains of people with a mental illness [36].

### Peer expertise

The expertise of people who experience(d) mental ill health can be regarded as a third domain of expertise, in addition to scientific evidence and practical knowledge and skills. Peer experts are the living example that recovery is possible and they can support other service users in their recovery journey [81–83]. The term ‘nothing about us without us’ is applied. Service users become equal partners in the design, delivery, steering and evaluation of a service [84].

In Europe lived experience has been acknowledged more and more as a domain of expertise [81], as shown in international and European policy documents and strategies of European service providers [83]. However, this is not always reflected in practice as only a few peer experts are paid and there is still a taboo on the self-disclosure of professionals [85].

To promote the involvement of service users as partners on policy, service and individual levels in the design and evaluation of services a lot of effort and organisation is needed to build peer expertise.

Firstly, this requires the empowerment of service users and their carers to take a step in building this expertise contributing to the mental health sector. This inherently means that power of the service providers is redistributed. Also, service users can only be empowered to take up their role as peer expert if there is community level understanding and acceptance about mental health and peer expertise.

Secondly, resources should be allocated to allow for the organisation of user-led services including representative organisations that give input to public and political activities and are active at the (inter-)national, regional and local levels, offering (ex-)service users the possibility to develop their expertise [86].

To improve the use of lived experience of professionals as a tool to support their clients, effort should be taken to break the taboo of self-disclosure [85].

Examples of good practices that can support the use of lived experience in organisations are training manuals for peer experts which have been developed nationally and internationally. An example of a European manual is the peer2peer vocational training course developed as part of the lifelong learning program funded by the EU [87]. A program that focuses on self-disclosure among professionals has been developed by ‘Samen Sterk Zonder Stigma’ [88].

## Conclusions

Discussion among European professional-, scientific- and peer- experts and members of the EUCOMS network resulted in an overview of six principles that serve as a foundation for a national, regional and local model of integrated mental health care. High quality community-based mental health care: 1) protects human rights; 2) has a public health focus; 3) supports service users in their recovery journey; 4) makes use of effective interventions based on evidence and client goals; 5) promotes a wide network of support in the community, and; 6) makes use of peer expertise in service design and delivery.

The six principles can be explained from three interrelated perspectives. The first combines the human rights and public health principle in the citizenship or societal perspective, that argues for the protection of human rights for all, including people with mental illness. The second is the personhood or the service user perspective, which combines the recovery and peer expertise principle. This perspective puts emphasis on the centrality of the service user in care and the use of their expertise in service design and provision. The last perspective is the quality of care or the professional perspective, combining the effectiveness of interventions and the community network of care principles. This last perspective argues that interventions are effective when they take into account local realities and work with the network, both formal and informal, of the service users.

In practice this means that services align their strategy with the CRPD and focus on population-based needs in the region. This asks for a versatile and efficient approach with a balance between community-based and hospital care [7, 89] where services, sectors, service users and their network closely collaborate [76]. This community network of care facilitates the personal recovery journey [90], and at the same time mitigates the effects of social inequality on mental health covering promotion, prevention, treatment and rehabilitation addressing the economic, social and physical environment in which people live [77]. Service providers have the task to use effective interventions adapted to reflect local realities. How the services are organised is context dependent and influenced by the culture, geography, the health system and the available financial resources [2, 89].

Although the authors could not find a similar paper outlining recommendations for the implementation of quality community-based mental health care, the outcomes of this article align with international policy documents [2, 91] and studies [89, 90, 92] that aim to define the characteristics of good mental health care. In line with the vision of EUCOMS members good mental health services are described as comprehensive, equally accessible, integrated, recovery oriented, aimed to protect and respect human rights, employ effective and tailored interventions, and work in collaboration with service users and his/her network.

Experience has learnt that governments and health care funders can support ‘good’ community-based mental health services through appropriate legislation and funding schemes. With this position paper EUCOMS hopes to contribute to the discussion on how to improve structures in mental healthcare, and to narrow the gap between evidence, policy and practice in Europe. Essential next steps for EUCOMS to succeed are to connect and involve the diverse stakeholder groups in ongoing dialogue, research consensus and capacity building, and advocacy.

### Limitations

Although the authors tried to develop a consensus based on the perspective of a broad variety of stakeholders both in terms of their role within the mental health system and country of origin, it must be noted that not all stakeholder groups have been equally represented. This article presents the view of EUCOMS members on what the principles and key-elements are of high-quality community-based mental health care. EUCOMS members are mental health service directors, umbrella organisation directors, mental health care professionals, peer experts, researchers and policy advisors who are mostly in favour of community-based mental health care, as they became member of the network to promote its implementation. In the development of the shared vision professionals from Western European countries were overrepresented. Relatively little service users and carers, and respondents from Southern and Eastern European countries provided input. This could have resulted in a view that does not sufficiently reflect counterarguments for community-based mental health care, the perspective of the service users and carers, and the socio-cultural and economic context in Southern and Eastern European countries.

## Data Availability

Not applicable – not datasets were generated or analysed.

## Abbreviations

ACT: Assertive Community Treatment
CBT: Cognitive Behavioural Therapy
CRPD: Convention on the Rights of Persons with Disabilities
EAOF: European Assertive Outreach Foundation
EFPC: The European Forum for Primary Care
EFQM: European Foundation for Quality Management
EUCOMS: European Community Mental Health Services Providers
F-ACT: Flexible Assertive Community Treatment
IDDT: Integrated Dual Disorder Treatment
IPS: Individual Placement and Support
MI: Motivational Interviewing
NICE: National Institute for health and Care Excellence
TLC3: Targeted, Local, Contact, Credible and Continuous
UDHR: Universal Declaration of Human Rights
UN: United Nations
WHO: World Health Association
